# Supplementary material for: Development and Validation of a Standardized Pseudotyped Virus-Based Neutralization Assay for Assessment of Anti-Nipah Virus Neutralizing Activity in Candidate Nipah Vaccines
Source: Vaccines (Basel). 2025 Jul 15;13(7):753. doi: 10.3390/vaccines13070753 (PMC12300343; doi:10.3390/vaccines13070753)
Supplement: Supplementary file 1 [file vaccines-13-00753-s001.zip › vaccines-3707902-supplementary.pdf]

<u>Supplementary data</u>

Table-S1: Panel of serum samples for Nipah pseudotyped virus neutralization assay validation

Serum ID	Source of serum
NV-1	WHO international standard (pool from 36 NiV convalescent individual)
NV-2	Bangladesh “high” pool (4 individuals)
NV-3	Bangladesh “low” pool (4 individuals)
NV-4	Bangladesh clinical sample (high)
NV-6	Malaysia clinical sample (low)
NV-10	Malaysia “mid” pool (3 individual)
NNV-1	2.67-fold diluted NV-1
NNV-2	5-fold diluted NV-1
NNV-3	2.5-fold diluted NV-1
NNV-4	2-fold diluted NV-1
NC-1	Bangladeshi healthy individuals
NC-2	Bangladeshi healthy individuals
NC-3	Bangladeshi healthy individuals
NC-4	Bangladeshi healthy individuals
NC-5	Bangladeshi healthy individuals

Table-S2: List of formulas used to Nipah pseudotyped virus neutralization assay validation

SL	Definition	Equation
(a)	MOI[a]	$2\ MOI\ = \frac{Number\ of\ cells}{Virus\ titer\ per\ \mu L} \times 2$
(b)	IU[b] conversion	$IU = \frac{Neutralizing\ antibody\ titer\ (NT50)\ of\ sample}{WHO\ IS^{c}\ neutralizing\ antibody\ titer\ in\ plate} \times 1000$
(c)	Relative accuracy	$Relative\ accuracy\ = \frac{GMT^{d}{}_{Observered}}{\mathrm{GMT}_{\mathrm{expected}}} \times 100\%$
(d)	Precision	$\%\ \mathrm{GCV}^{e} = (10^{standard\ deviation} - 1) \times 100\%$
(e)	LLOQ[f]	$LLOQ = \frac{Standard\ deviation}{Slope} \times 10$

a MOI, Multiplicity of infection
b IU, International Unit
c WHO IS, WHO International Standard
d GMT, Geometric mean titer
e GCV, Geometric coefficient of variation
f LLOQ, Lower limit of quantification

Table-S3: Comparative analysis of inter-laboratory specificity for anti-NiV neutralizing antibody detection:

(a) Correlation of 5 positive samples between MHRA (reference study) and icddr,b (new) laboratories

Serum ID	Reference titer (IU/mL)	New titer (IU/mL)	Status (Ref / new)	Ratio (Ref / New)
**NV-2**	654	1019	(Pos / Pos)	0.64
**NV-3**	113	121	(Pos / Pos)	0.93
**NV-4**	1218	1074	(Pos / Pos)	1.13
**NV-6**	159	151	(Pos / Pos)	1.05
**NV-10**	252	257	(Pos / Pos)	0.98

**Geometric mean ratio (GMR)**	**0.93**
**Observed range**	0.64 – 1.13

(b) Evaluation of 5 negative samples within icddr,b based on ELISA (reference) and NiV-PNA (new) results

Serum ID	Reference titer (IU/mL)	New titer (IU/mL)	Status (Ref /New)	Ratio (Ref / New)
**NC-1**	Negative	<2.78	(Neg / Neg)	N/A
**NC-2**	Negative	<2.78	(Neg / Neg)	N/A
**NC-3**	Negative	<2.78	(Neg / Neg)	N/A
**NC-4**	Negative	<2.78	(Neg / Neg)	N/A
**NC-5**	Negative	<2.78	(Neg / Neg)	N/A

Table-S4: Evaluation of dilutional linearity and relative accuracy using dilution series (1:1 (neat) to 1:32) of three anti-NiV neutralizing antibody positive serum samples (NV-02, NV-04, and NV-10)

Sample ID	Dilution factor	Measured titer (IU/mL)	Expected titer (IU/mL)	Relative accuracy (%) (Measured titer/ Expected titer)
**NV-2**	1	762	762	100.00
	2	375	381	98.44
	4	153	190	80.46
	8	82	95	86.64
	16	46	48	95.59
	32	22	24	91.63
**NV-4**	1	1728	1728	100.00
	2	934	864	108.10
	4	555	432	128.47
	8	260	216	120.24
	16	126	108	116.62
	32	54	54	99.70
**NV-10**	1	336	336	100.00
	2	172	168	102.17
	4	81	84	95.88
	8	32	42	76.90
	16	20	21	97.21
	32	9	11	84.09
**Geometric mean (GM)**				98.18
**Observed range**				76.90 – 128.47
**Acceptable range**				70.00 – 130.00

Table-S5: For precision assessment: a panel of 10 serum samples covering negative, low, intermediate, and high anti-NiV neutralization antibody titer was tested in duplicate by two analysts over three days. Samples NC-1, NC-2 were not included in the calculations, as both samples were negative.

Sample ID	Day	Analyst -1 (IU/mL)		Analyst -2 (IU/mL)	
		Replicate-1	Replicate-2	Replicate-1	Replicate-2
**NV-2**	Day-1	586	647	631	714
	Day-2	596	768	514	599
	Day-3	494	621	478	543
**NV-4**	Day-1	933	1142	1088	1174
	Day-2	891	1073	939	924
	Day-3	1105	1245	808	955
**NV-6**	Day-1	151	152	129	108
	Day-2	123	159	182	136
	Day-3	155	158	134	133
**NV-10**	Day-1	265	228	208	210
	Day-2	274	291	248	291
	Day-3	268	244	198	176
**NNV-1**	Day-1	284	290	250	267
	Day-2	321	264	219	286
	Day-3	196	263	223	183
**NNV-2**	Day-1	136	125	155	129
	Day-2	127	137	123	117
	Day-3	146	118	99	77
**NNV-3**	Day-1	279	331	276	214
	Day-2	316	377	321	307
	Day-3	479	360	223	237
**NNV-4**	Day-1	701	740	601	517
	Day-2	886	755	767	768
	Day-3	661	672	995	772
**NC-1**	Day-1	Negative	Negative	Negative	Negative
	Day-2	Negative	Negative	Negative	Negative
	Day-3	Negative	Negative	Negative	Negative
**NC-2**	Day-1	Negative	Negative	Negative	Negative
	Day-2	Negative	Negative	Negative	Negative
	Day-3	Negative	Negative	Negative	Negative

Table-S6: The LLOQ[g] was determined by generating a calibration curve using NV-04, a positive human serum sample for anti-NiV neutralizing antibodies. The LLOQ for neutralizing anti-NiV antibodies in human serum was calculated based on the standard deviation of the response and the slope of the calibration curve.

Sample ID	Dilution factor	Observed titer (IU/mL)	Expected titer (IU/mL)	Expected titer (log10)	Observed titer (log10)
NV-4	1	1209	1209	3.08	3.08
	2	610	605	2.78	2.79
	4	301	302	2.48	2.48
	8	201	151	2.18	2.30
	16	100	76	1.88	2.00
	32	43	38	1.58	1.63
	64	22	19	1.28	1.34
	128	10	9	0.98	0.99
	256	5	5	0.67	0.69
	512	3	2	0.37	0.41
	1024	1	1	0.07	0.08
	2048	1	1	-0.23	-0.19
	4096	0	0	-0.53	N/A

**Standard deviation**	0.04
**Geomean**	3.13
**Slope**	1.0007
**log (LLOQ)**	0.44
**LLOQ (IU/mL)**	**2.78**

[g] LLOQ, Lower limit of quantification

Table-S7: Evaluation of assay robustness using three anti-NiV antibody-positive serum samples. Sample variability was evaluated using the %GCV.

(a) Performance comparison between two different NiV-PsV lots (Lot-1 and Lot-2) on assay results.

Sample ID	PsV Lot-1	PsV Lot-2	Geomean	SD[h]	GCV[i]
**NV-2**	2.78	2.79	2.79	0.01	1.26%
**NV-4**	3.00	3.02	3.01	0.02	4.12%
**NNV-4**	2.94	3.01	2.97	0.05	12.63%
		**Geomean of GCV**			4.03%

(b) Impact of varying Vero cell numbers per well (15000, 18000, 20000, 22000, and 25000) on assay performance.

Sample ID	15k	18k	20k	22k	25k	Geomean	SD	GCV
**NV-2**	2.51	2.65	2.89	2.70	2.47	2.64	0.17	46.66%
**NV-4**	3.00	3.03	3.02	3.07	2.87	3.00	0.07	18.39%
**NNV-4**	2.89	3.07	3.03	3.01	2.85	2.97	0.09	24.42%
			**Geomean of GCV**					27.57%

(c) Effect of different incubation periods on day 2 of the NiV-PNA (18, 20, 22, and 24 hours) on assay outcomes.

Sample ID	24hr	22hr	20hr	18hr	Geomean	SD	GCV
**NV-2**	2.71	2.78	2.89	2.67	2.76	0.10	24.50%
**NV-4**	2.91	2.95	3.02	2.84	2.93	0.07	18.45%
**NNV-4**	2.93	2.97	3.04	2.84	2.95	0.08	20.20%
					Geomean of GCV		20.90%

[h] SD, Standard deviation
[i] GCV, Geometric coefficient of variation

	1	2	3	4	5	6	7	8	9	10	11	12
A	PBS	PBS	PBS	PBS	PBS	PBS	PBS	PBS	PBS	PBS	PBS	PBS
B	PBS	NiV-PsV									CC	PBS
C	PBS	NiV-PsV									CC	PBS
D	PBS	NiV-PsV									CC	PBS
E	PBS	NiV-PsV									CC	PBS
F	PBS	NiV-PsV									CC	PBS
G	PBS	NiV-PsV									CC	PBS
H	PBS	PBS	PBS	PBS	PBS	PBS	PBS	PBS	PBS	PBS	PBS	PBS

5-fold serial dilution

CC = Cell control, PBS = Phosphate buffer saline, NiV-PsV= Initial diluted Nipah pseudotyped virus

Figure-S1: Microplate layout- Nipah pseudotyped virus $TCID_{50}$ determination

	Sample 1		Sample 2		Sample 3		Sample 4		PC 1		PC 2	
	1	2	3	4	5	6	7	8	9	10	11	12
A												
B												
C												
D												
E												
F												
G												
H	CC	CC	CC	CC	VC	VC	VC	VC	VC	VC	VC	VC

CC = Cell control, VC = Pseudotyped virus control, PC = Positive serum control

Figure-S2: Microplate layout- NiV-PNA in Vero cells
